# Effects of selective stimulation of apical electrodes on temporal pitch perception by cochlear implant recipients

**DOI:** 10.1121/10.0029023

**Published:** 2024-09-30

**Authors:** Evelien De Groote, Robert P. Carlyon, John M. Deeks, Olivier Macherey

**Affiliations:** 1 Cambridge Hearing Group, Medical Research Council Cognition and Brain Sciences Unit, University of Cambridge, Cambridge, CB2 7EF, United Kingdom; 2 Aix Marseille Université, Centre National de la Recherche Scientifique, Centrale Méditerranée, Laboratoire de Mécanique et d'Acoustique, Centre National de la Recherche Scientifique, Aix Marseille Université, Marseille, 13453 Cedex 13, France

## Abstract

This study investigated whether selective apical stimulation improves temporal pitch perception in eight MED-EL cochlear implant recipients and whether any such improvement relates to auditory-nerve survival. Three stimulation conditions differing in the place and width of excitation were evaluated: single-electrode stimulation of (i) the most apical, (ii) a mid-array electrode, and (iii) multi-electrode stimulation of the four most apical electrodes. Stimulation-current-induced non-stimulating electrode voltages were recorded to identify extracochlear electrodes and gauge insertion depth. The pitches of the four most apical electrodes were compared using place-pitch ranking. Rate-pitch ranking was assessed between 80 and 981 pulses per second for the three stimulation conditions, to estimate the “upper limit” of temporal pitch. Single-electrode apical stimulation did not increase the upper limit relative to other conditions. The polarity effect (PE), defined as the difference between thresholds obtained for triphasic pulse trains with their central high-amplitude phase either anodic or cathodic, was obtained to evaluate peripheral neural health. The PE did not differ between apical and mid-array stimulation or correlate with the upper limit. In conclusion, we found no improvement of temporal pitch perception with single-electrode apical stimulation, and discuss possible explanations for this observation.

## I. INTRODUCTION

Cochlear implants (CI) convey pitch cues along two independent perceptual dimensions corresponding to the place and temporal pattern of stimulation (Macherey *et al.*, 2011; McKay *et al.*, 2000; Tong *et al.*, 1983). However, both suffer from distinct limitations that may contribute to the difficulties that CI recipients experience with speech perception in noisy backgrounds (Arenberg *et al.*, 2018; Luo *et al.*, 2021). First, the range of place-pitch cues is limited by insertion depths that usually do not extend all the way into the apex of the cochlea (Boyd, 2011), as well as by the restricted number of implantable electrodes and broad current spread (Shannon *et al.*, 1995). Second, the perception of temporal pitch cues in CI recipients is surprisingly poor compared to normal-hearing listeners for reasons that remain incompletely understood. For pulse trains presented on a single electrode, CI recipients typically report increasing pitch with increasing pulse rates up to about 300 to 500 pulses per second (pps), above which increases in pulse rate do not elicit reliable pitch changes (Kong *et al.*, 2009; Townshend *et al.*, 1987; Zeng, 2002). This “upper limit of temporal pitch perception” is substantially lower than that usually found in normal-hearing (NH) listeners, even when using stimuli that do not contain reliable place-pitch cues (Carlyon and Deeks, 2002; Macherey and Carlyon, 2014). Moreover, even at pulse rates below this upper limit, temporal pitch sensitivity is typically worse than for NH listeners (Stahl *et al.*, 2016). Three possible reasons for the poor temporal pitch perception are described briefly in the following, namely, limitations in stimulating sufficiently apically, in stimulating sufficiently selectively, and in neural health. We then present a summary of the experiments we performed to investigate the role of each limitation.

### A. Apical stimulation

Middlebrooks and Snyder (2010) recorded phase-locking of neurons in the cat's inferior colliculus (IC) in response to electrical stimulation of the auditory nerve (AN) and found that the highest pulse rate to which IC neurons phase-locked was greatest when stimulation was selective (i.e., minimal spread of excitation) and originated from neurons innervating the apex compared to other cochlear sites. They argued for the existence of a specialized low-frequency brainstem pathway that supports precise temporal processing and further suggested that selective stimulation of this pathway might improve temporal acuity in human CI recipients.

A number of studies have investigated whether temporal pitch perception in human CI recipients does indeed vary systematically along the electrode array using direct-stimulation experiments. The majority of these studies have not found a significant effect of electrode location on various outcome measures of rate-pitch perception (Baumann and Nobbe, 2004; Goldsworthy and Shannon, 2014; Ihlefeld *et al.*, 2015; Kong *et al.*, 2009), although substantial within-subject variations have been noted (Ihlefeld *et al.*, 2015). One possible reason for these null findings may be that the most apical electrode tested in these studies was not apical enough. Only three studies (Baumann and Nobbe, 2004; Kong *et al.*, 2009; Stahl *et al.*, 2016) included CI recipients with MED-EL devices, whose electrode arrays allow for a deep insertion into the apex of the cochlea. MED-EL's longest electrode arrays, the Standard and FLEX28, measure 31.5 and 28 mm and have been reported to reach average insertion angles of up to 700 and 471 degrees, respectively (Landsberger *et al.*, 2015; Trieger *et al.*, 2011), whereas typical insertion angles vary between 350 and 480 degrees for other manufacturers (Landsberger *et al.*, 2015). With such high insertion angles, the most apical electrode of the Standard and FLEX28 array can, in principle, stimulate AN fibers tuned to much lower characteristic frequencies (Landsberger *et al.*, 2015). Both Baumann and Nobbe (2004) and Kong *et al.* (2009) found no effect of apical stimulation, but the most-apical electrode was e3, which may not have reached the low-frequency AN fibers that are believed to support precise temporal processing. Furthermore, and with the exception of Kong *et al.* (2009) who report a full insertion of the electrode array up to 30 mm into the cochlea, none of the previously noted studies reports on insertion depth, casting even more doubt on truly how apically these were stimulating. Another reason is that place-pitch perception (i.e., pitch judgements of sounds produced by different electrodes) at the apex of the MED-EL array specifically is sometimes poor and can show pitch reversals (Baumann and Nobbe, 2006; Gani *et al.*, 2007; Kenway *et al.*, 2015). In contrast to studies reporting null findings, Stahl *et al.* (2016) observed significantly lower rate-discrimination thresholds for pulse rates between 20 and 104 pps when effectively selecting the apical electrode that produced the lowest place pitch and comparing it to a basal electrode.

Two other studies used a different approach to examine the benefit of apical stimulation on temporal pitch perception. Macherey *et al.* (2011) and Lamping *et al.* (2020) both stimulated an electrode pair at the apex of the cochlea with pseudomonophasic pulses presented in a narrow bipolar stimulation mode. They predicted that the place of excitation could be shifted more apically when the first high-amplitude phase of the pseudomonophasic pulse was anodic on the more apical electrode of the pair compared to when it was cathodic. This prediction was confirmed in a place-pitch ranking experiment, which showed a significantly lower pitch rank for anodic-dominant compared to cathodic-dominant stimulation and suggests that the anodic-dominant stimulation reached AN fibers with lower characteristic frequencies. In line with the hypothesis of Middlebrooks and Snyder (2010), the combined data from the two studies revealed a significantly higher upper limit of temporal pitch perception for anodic-dominant compared to cathodic-dominant stimulation (Lamping *et al.*, 2020), consistent with apical stimulation improving temporal pitch perception.

Altogether, there is some evidence that apical stimulation can improve temporal pitch perception by CI recipients provided that the place of stimulation is sufficiently apical. There are several methods to infer electrode positions (e.g., medical imaging, impedance data, pitch ranking) and confirm that the most apical electrode is inserted deeply into the cochlea, where we expect temporal pitch processing to be superior.

### B. Selective stimulation

It may be that accurate temporal processing of pitch requires that stimulation is not only apical but also selective (Middlebrooks and Snyder, 2010). In this regard, Macherey *et al.* (2011) note that the temporal information coming from apical AN fibers may be “blurred” by more basal fibers projecting to the IC neurons with a lower upper limit of phase locking. If this is true, then effectively broadening the excitation pattern by stimulating multiple electrodes at the same time could potentially degrade temporal pitch perception. Alternatively, other authors have argued that broadening the excitation pattern might *improve* temporal pitch perception, either by increasing the number of neurons conveying temporal information (allowing for “multiple looks”) or by increasing the chances of stimulating a cochlear region with accurate temporal pitch processing (Kong *et al.*, 2009; Penninger *et al.*, 2015).

With one exception, studies that have compared temporal pitch perception for single- and multi-electrode stimulation used interleaved rather than truly simultaneous stimulation of multiple electrodes (Carlyon *et al.*, 2010; Penninger *et al.*, 2015; Venter and Hanekom, 2014). Interleaved stimulation can complicate the interpretation of the results, because each neuron will be excited by multiple pulses from different electrodes, leading to a complex pattern of stimulation. The one study that did use truly simultaneous stimulation found no significant overall difference between single- and multi-electrode stimulation of electrodes at the middle of a MED-EL array on rate-discrimination thresholds for pulse trains between 100 and 566 pps, but with some evidence that a subgroup of nine well-performing participants performed better in the multi-electrode condition (Bahmer and Baumann, 2013).

Overall, a reasonable conclusion is that broadening the excitation pattern through stimulation of multiple non-apical electrodes does not lead to consistent changes in rate-pitch encoding by CI recipients. However, none of these studies have specifically considered the effect of simultaneously stimulating multiple electrodes at the apex, where we expect selectivity to be of importance to target the AN fibers with low characteristic frequencies (Middlebrooks and Snyder, 2010).

### C. Neural health

Although rate-pitch perception does not seem to vary systematically with stimulation site or width of excitation in CI recipients, one common finding is that performance across electrodes can vary idiosyncratically and reliably for a given participant (Ihlefeld *et al.*, 2015; Cosentino *et al.*, 2016). This observation has raised the question of whether across-electrode variations of temporal pitch perception could be related to variations in neural health. One aspect of neural health that may vary along the electrode array is the survival of the peripheral AN processes, which typically degenerate before the central axon after deafness (Nadol, 1990; Schuknecht, 1993). This may be especially important for stimulation of the cochlear apex, due to the fact that Rosenthal's canal, which contains the cell bodies of the spiral ganglion neurons, does not extend all the way into the apex. Hence, in the absence of peripheral processes, electrodes located deep into the apex may excite distant and tightly packed cell bodies or central axons (Ariyasu *et al.*, 1989; Spoendlin and Schrott, 1989; Stakhovskaya *et al.*, 2007). Computational models predict that cathodic pulse trains will preferentially stimulate the peripheral processes of the spiral ganglion cells, whereas anodic stimulation preferentially activates the central axon (Rattay *et al.*, 2001; Resnick *et al.*, 2018). Hence, differences between the sensitivity to asymmetric pulses in which the highest-amplitude phase is cathodic vs anodic may serve as an indicator of the survival of peripheral processes of the AN (but see Kalkman *et al.*, 2022). To assess whether the benefit of selective apical stimulation on temporal pitch perception relies on good peripheral neural health, the present study measured this “polarity effect” (PE) by measuring detection thresholds for trains of triphasic pulses of different polarity (cf. Carlyon *et al.*, 2018; Macherey *et al.*, 2017; Mesnildrey *et al.*, 2020). Several studies have shown that the PE obtained with such asymmetric pulse shapes correlates positively, across electrodes, with the average of the thresholds obtained with the two polarities or with the detection threshold for a symmetric pulse. As detection thresholds have been considered a potential correlate of neural degeneration (with higher thresholds indicating more advanced neural degeneration), its relationship with the PE has been taken as evidence that the PE does indeed reflect the survival of the peripheral processes (Carlyon *et al.*, 2018; Jahn and Arenberg 2019a,b; Mesnildrey *et al.*, 2020; Brochier *et al.*, 2021). However, Kalkman e*t al.* (2022) have argued that this correlation may be obtained without recourse to neural survival, and we will return to this argument in the Discussion.

### D. Study overview

The present study investigates temporal pitch perception in three stimulation conditions that one would reasonably expect to affect either the place of stimulation or the spread of excitation. Specifically, rate-pitch ranking for (i) single-electrode apical stimulation was compared to (ii) single-electrode stimulation of a mid-array electrode, and (iii) simultaneous multi-electrode apical stimulation. We tested recipients of the MED-EL implant, which has a longer electrode array and has the potential of stimulating more apically than other devices, provided that the electrode array fully covers the cochlea. To address this issue, stimulation-current-induced non-stimulating electrode voltage (SCINSEV) recordings were measured as a proxy of insertion depth. In addition, we performed place-pitch ranking at the apex to ensure that the most apical electrode effectively produced the lowest pitch percept. If accurate temporal pitch processing relies on a specialized low-frequency brainstem pathway that can be selectively stimulated, we expected higher upper limits and steeper high-rate slopes for single-electrode stimulation of the most apical electrode compared to stimulation conditions that affect either the place (i.e., non-apical) and the width (multi-electrode) of stimulation. Moreover, since it is assumed that selective stimulation at the apex depends on the presence of the peripheral AN processes, we hypothesized that those participants with good apical neural health as estimated by the PE would show the greatest improvements in temporal pitch perception with selective apical stimulation compared to other stimulation conditions.

## II. METHODS

### A. Participants

Eight participants [two women and six men, mean age 70 ± 10.7 standard deviation (*SD*) years] took part in this two-session study. Both sessions took about 3 h to complete and were performed on two separate days by most participants. The time between sessions ranged from 3 to 15 days. Only participants M020 and M030 did the two sessions on the same day. All participants were postlingually deafened and had been using their MED-EL device for at least 2 (mean 8 ± 4.1 *SD*) years at the time of the experiment. The majority of participants were bilaterally implanted, and in these, the earliest-implanted side was tested. All participants were using one of MED-EL's fine-structure stimulation strategies in their clinical processing strategy, which aims to provide temporal fine structure information at the four most apical electrodes e1–e4 (Dhanasingh and Hochmair, 2021). MED-EL's electrode arrays contain 12 electrodes that are numbered from 1 to 12 from apex to base. Table I shows participants' demographic and hearing-related characteristics, as well as their device specifications.

**TABLE I.** Details of participants.

Subject	Age (yr)	Sex	Etiology	Duration of hearing loss^a^ (yr)	Implant experience (yr)	Implanted side	Array type	Array length (mm)	Electrode spacing (mm)	Deactivated electrodes	Extracochlear electrodes	Max. insertion depth (mm)
M004	83	M	Unknown	10	15	Right	Standard	31.5	2.4	/	2	24.0
M013	81	F	Unknown	1	10	Left	Standard	31.5	2.4	e10, e11, e12	2	24.0
M014	67	M	Autoimmune disease	1	10	Right	FLEX28	28.0	2.1	/	0	27.1
M020	79	M	Trauma	3	10	Left	Standard	31.5	2.4	e9, e10, e11, e12	4	19.2
M030	53	F	Otosclerosis	20	6	Right	FLEX28	28.0	2.1	e12	1	23.1
M031	74	M	Ototoxicity	11	7	Left	Standard	31.5	2.4	e12	2	24.0
M032	62	M	Temporal bone fracture	1	4	Right	FLEX28	28.0	2.1	/	1	23.1
M035	63	M	Meniere	28	12	Left	FLEXSOFT	31.5	2.4	/	0	30.4

^a^ The duration of hearing loss refers to the pre-implantation period. The maximum insertion depth (mm) refers to the deepest possible insertion depth of the most apical electrode assuming a “best-case scenario” for all participants.

During the experiments, participants replaced their external speech processor with a coil connected to a computer via the MAX Programming Interface System (MED-EL, Innsbruck). Stimulus presentation was controlled via interfaces programmed in matlab R2020b (Mathworks, Natick, MA) that used low-level routines provided by MED-EL. Prior to this study, all stimuli were checked using a digital oscilloscope.

All participants signed the informed consent and were compensated for their time. The study was approved by the Committee for the Protection of Persons (CPP) Sud-Est 1 (ID RCB 2022-A01600-43).

### B. Impedances and induced voltage recordings

**FIG. 1.**
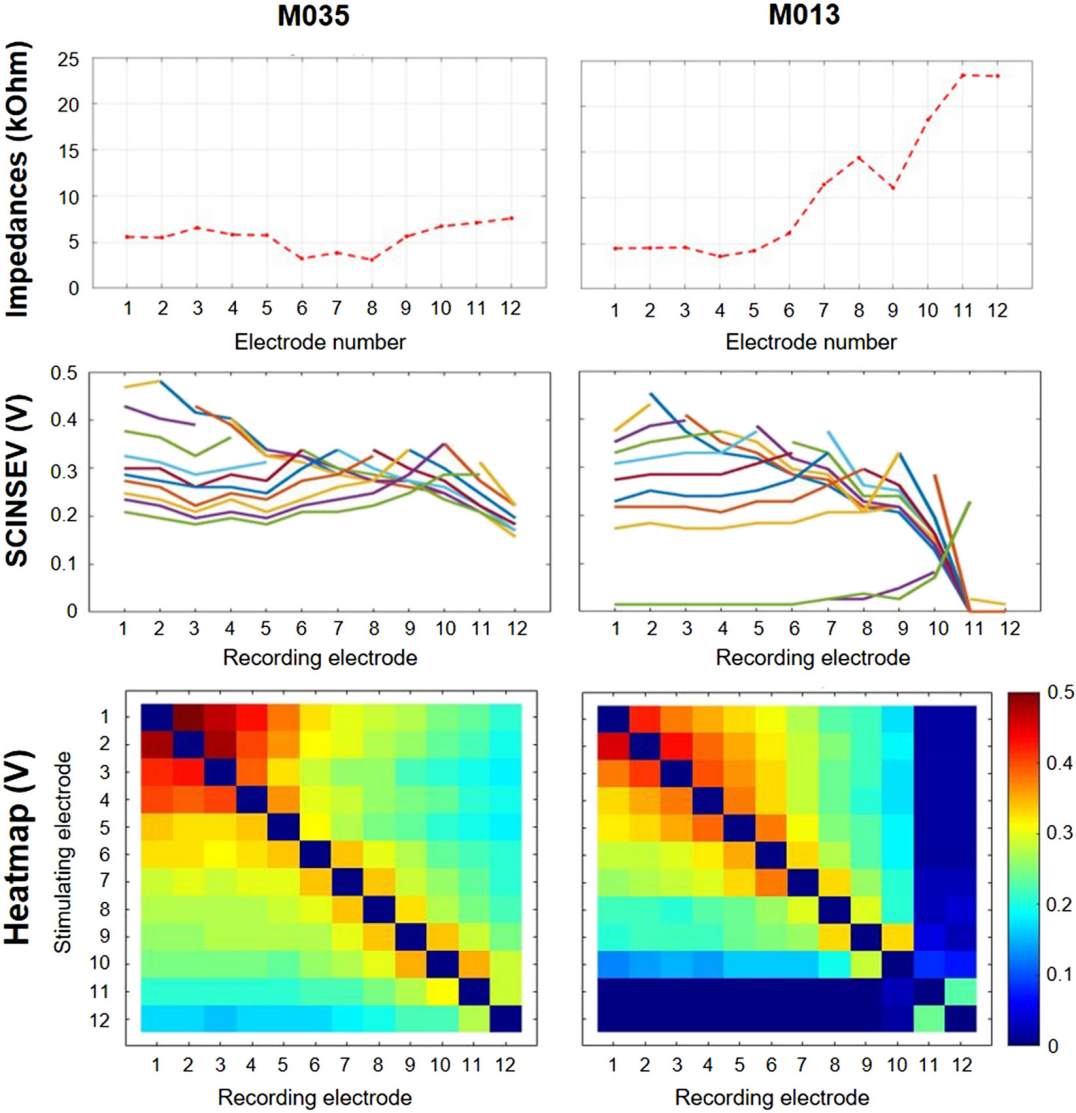
(Color online) Impedance and Field Telemetry measurements taken with the clinical fitting software MAESTRO 9.0 for participant M035 (left panel) and participant M013 (right panel). Electrodes are always numbered apical (1) to basal (12). Top panels show contact impedances for individual electrodes. Middle and bottom panels show SCINSEV recordings presented as lineplots and heatmaps, respectively. The colored lines in the lineplots represent the different stimulating electrodes. SCINSEV recordings were used to estimate the number of extracochlear electrodes by two experienced senior audiologists. Participant M035 was judged to have 0 extracochlear electrodes, whereas participant M013 was judged to have two extracochlear electrodes. This is evident from (1) high contact impedances at the two most basal electrodes, (2) the change in slopes and clustering at the skirts for these electrodes as can be observed from the lineplots, and (3) the heatmap, which shows a rapid drop of the voltage for these electrodes while other electrodes show more gradually decreasing voltages.

At the beginning and end of each session, a series of measures were taken with MED-EL's clinical fitting software MAESTRO 9.0 using the Impedance and Field Telemetry module. Contact impedances as well as induced voltage recordings were measured by stimulating an electrode pair and measuring the resulting voltage from all (both stimulating and non-stimulating) electrodes in the array relative to the extracochlear return electrode. The electrical stimuli were cathodic-leading biphasic pulses with an amplitude of 300 *μ*A, phase duration of 24 *μ*s, and inter-phase gap of 2.1 *μ*s. Contact impedances (kΩ) were checked to ensure that all stimulation levels were within the compliance levels of the device. SCINSEV recordings were measured to assess the longitudinal electrical spread along the electrode array and the transverse electrical spread out of the cochlea (de Rijk *et al.*, 2020). SCINSEVs have proven useful for the detection of individual electrodes on the array located outside of the cochlea due to incomplete insertion or extrusion (i.e., extracochlear electrodes). As the current study probed the effect of apical stimulation on temporal pitch perception, and we were unable to access post-operative computed tomography (CT) scans, the presence and number of extracochlear electrodes were used as a proxy of the insertion depth of the electrode array, with a greater number of extracochlear electrodes necessarily limiting insertion depth. Voltage responses for the stimulating electrodes were plotted as a function of the recording electrode by means of line plots and heatmaps. Two senior audiologists with extensive experience in SCINSEV recordings were asked to independently judge the number of extracochlear electrodes based on the data recorded at the beginning and end of the first session (*N* = 16, 8 CI recipients × 2 measurements). In case of inconsistencies, individual cases were discussed until a consensus was reached. As an example, Fig. 1 presents two participants who were judged to have complete (M035) and incomplete (M013) insertions.

The estimated number of extracochlear electrodes was then used to gauge the maximum absolute insertion depth (mm) of the most apical electrode assuming the “best-case scenario” for all participants. For participants with complete insertions, maximum insertion depth was calculated based on the assumption that the stopper marker on the electrode array is located at the round window. For participants with incomplete insertions, maximum insertion depth was calculated based on the assumption that the lowest-numbered extracochlear electrode was located just outside the round window.

### C. Polarity effect

Electrical stimuli were 400-ms pulse trains presented at a rate of 99 pps. Triphasic pulses were used, in which the amplitude of the central phase was twice that of each of the first and third phases. The polarity of the central high-amplitude phase could be either anodic or cathodic, respectively referred to as TP-A and TP-C (Carlyon *et al.*, 2013; Macherey *et al.*, 2006). Phase duration was 40 *μ*s, and the inter-phase gap was set to 0 *μ*s. Each participant was tested on the same two electrodes that were chosen for the single-electrode apical (e1) and mid-array (e7 or e8) stimulation condition. The PE for the two electrodes was measured in separate sessions and in random order. All stimuli were presented in monopolar stimulation mode.

Initially, a loudness-scaling procedure was performed for each combination of participant and polarity, so as to guide the starting level and set a safety limit for the threshold measurements. Next, detection thresholds were measured using a three-down, one-up, two-interval forced-choice adaptive procedure that converged on 79% correct (Levitt, 1971). Participants indicated which interval contained the sound and responded by selecting the button of their choice on a computer screen. Trial-by-trial feedback was provided. The signal starting level was set to the level corresponding to “4 – Comfortable but Soft.” The step size was 1 dB for the first two reversals and 0.25 dB thereafter. The mean of the last six out of eight reversals was used to estimate the threshold. For most participants, four repetitions were performed for each measurement with TP-A and TP-C presented alternately, and with the starting polarity randomly chosen. Due to time constraints, only two repetitions were done for each polarity for participants M004 on both electrodes and M020 for the mid-array electrode. Thresholds were not measured at the mid-array electrode for participant M014, also due to time constraints. The PE was quantified by subtracting the TP-A from the TP-C thresholds (TP-C–TP-A). As a result, negative values indicate that the TP-C threshold was lower than the TP-A threshold, as would be expected from good survival of the peripheral processes.

Statistical analyses were run using IBM SPSS Statistics 28.0.0 (IBM Corp., Armonk, NY). First, to establish whether the previously observed positive correlation between the PE and the average threshold (see Introduction) held in the present study, Pearson's correlation coefficients *r* were calculated between the average threshold [(TP-A+TP-C)/2] and the PE after removing between-participants differences (Carlyon *et al.*, 2018). This was done by subtracting from each individual data point of a given participant the average value across the two electrode locations for the same participant, leaving only the between-electrode correlation (e.g., for participant M032, the PE at the apical and mid-array electrodes was 0.69 and 1.04 dB, respectively, which equals –0.18 and 0.18 dB after normalization). Pearson's correlation coefficient cut-offs for small, medium, and large effects are generally set to 0.10, 0.30, and 0.50 (Cohen, 1992). Next, a paired-samples *t*-test was used to evaluate the difference between the PE measured at the apex and at the middle of the array, and effect sizes and their confidence intervals were calculated using Cohen's *d*. Cohen (1992) classifies effect sizes *d* as small (*d* = 0.20), medium (*d* = 0.50), and large (*d* ≥ 0.80).

### D. Place-pitch ranking

Place-pitch ranking was carried out to verify the place pitch produced by the four most apical electrodes and to check for monotonically increasing pitch with increasing electrode number. Electrical stimuli were 400-ms pulse trains presented at a rate of 80 pps. This low pulse rate was chosen to avoid any influence of between-electrode variations in the salience of temporal pitch on place-pitch judgements, as could occur with higher pulse rates due to variations in the upper limit of temporal pitch across electrodes. Biphasic pulses consisted of a 40-*μ*s cathodic phase, followed by a 2.1-*μ*s inter-phase gap and by a 40-*μ*s equal-amplitude anodic phase. All stimuli were presented in monopolar stimulation mode.

First, most comfortable levels (MCLs) were determined on individual electrodes (from e1 to e4) for each participant using loudness scaling. Participants indicated the loudness of stimuli on a chart with loudness marked on a scale from “0 – No audible sound” to “10 – Too loud.” Participants were asked to indicate “1 – Just noticeable” at the first instance they heard a sound and to indicate their perceived loudness for each subsequent sound. Stimulus levels were increased until the level corresponding to a perceived loudness of “7 – Comfortable but Loud” was reached, after which the stimulus level was decreased. All participant responses were recorded. The MCL was defined as the midpoint of stimulus levels that the participant indicated as “6 – Most Comfortable.” This procedure yielded an equal-loudness MCL profile across the four most apical electrodes for an 80-pps pulse train for each participant.

After loudness scaling, place-pitch ranking was assessed using the optimally efficient MidPoint Comparison (MPC, Long *et al.*, 2005) procedure. In the standard version of this procedure, participants make a series of two-interval forced-choice comparisons (“Which sound has the highest pitch?”) without feedback: the two stimuli to be compared on each trial are selected based on the results of the previous trial so as to minimize the total number of comparisons. The inter-stimulus interval was 800 ms. We used the “best-of-three” modification implemented by Adel *et al.* (2019) after Levitt and Rabiner (1967), in which comparisons are presented twice, with the order of the stimuli randomized each time. Participant responses in those two trials were then compared. In case of consistent responses on both trials, the procedure continued with the next comparison. In case of inconsistent responses on both trials, a third comparison was presented and the trial was scored according to the stimulus judged higher on two out of the three trials. This procedure was performed five times (or ten times for participant M004), each with the stimuli presented in a different random order. For each electrode, the mean pitch rank and *SD* were calculated across those runs.

The accuracy of apical place-pitch encoding was defined as the electrode that produced a pitch rank one *SD* above the mean rank of the most apical electrode (e1, Cosentino *et al.*, 2016). As such, the higher the electrode number, the poorer apical place-pitch encoding.

### E. Rate-pitch ranking

**FIG. 2.**
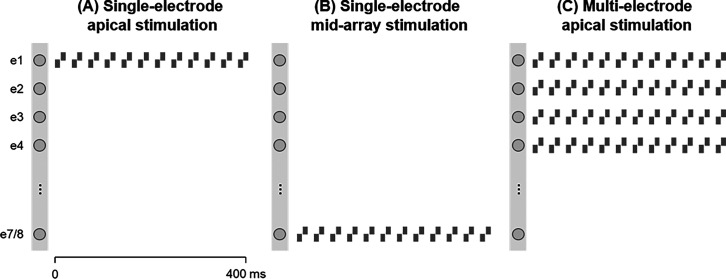
Schematic representation of the three stimulation conditions. During the single-electrode stimulation conditions, a pulse train was presented to either the most apical e1 (A) or a mid-array e7/e8 (B) electrode. During the multi-electrode stimulation condition, the four most apical electrodes e1-e4 were stimulated simultaneously (C). All stimulation conditions used biphasic cathodic-leading pulses with a 40-*μ*s phase duration and a 2.1-*μ*s inter-phase gap.

Rate-pitch ranking was carried out in three conditions: (i) single-electrode stimulation of the most apical electrode (e1), (ii) single-electrode mid-array stimulation (e7 or e8), and (iii) multi-electrode stimulation of the four most apical electrodes simultaneously (e1–e4). The three stimulation conditions are visualized in Fig. 2. Participants ranked eight 400-ms pulse trains, with their rates equally spaced on a logarithmic scale between 80 and 981 pps (i.e., 35% difference between consecutive rates). Biphasic pulses consisted of a 40-*μ*s cathodic phase, followed by a 2.1-*μ*s inter-phase gap and by a 40-*μ*s equal-amplitude anodic phase. All stimuli were presented in monopolar stimulation mode.

First, MCLs were estimated for 80-, 234-, 479-, and 981-pps pulse trains for each of the three stimulation conditions using the same loudness-scaling procedure as described previously. For each stimulation condition, individual pulse rates were then loudness-balanced by presenting them in pairs and using an adjustment paradigm. (Note that stimulation conditions were not loudness-balanced against each other.) The level of the first sound was always fixed at MCL, whereas the start level of the second sound was randomly set to the level corresponding to loudness “4 – Comfortable but too Soft” or “7 – Loud but Comfortable.” Different start levels were used on different runs to discourage participants from repeating their responses. Participants could adjust the level of the second sound by pressing one of six buttons labelled “-,” “–,” and “—” to make the second sound increasingly softer and “+,” “++,” and “+++” to make the second sound increasingly louder. The different buttons corresponded to 1-, 3-, and 6-bit steps [1 bit corresponds to current steps of 1.18, 2.36, 7.71, and 9.45 *μ*A for ranges 0 (0–150 *μ*A), 1 (151–300 *μ*A), 2 (301–600 *μ*A), and 3 (601–1200 *μ*A), respectively]. Each time one of the buttons was pressed, the stimulus pair was presented again until the two sounds were judged as equally loud. For each combination of condition and rate, loudness balancing was done four times (with the order of fixed and adjustable sound switched halfway). After loudness-balancing MCLs, levels in dB for the remaining intermediate pulse rates (114, 164, 335, and 686 pps) were linearly interpolated. Importantly, for the simultaneous multi-electrode stimulation of the four most apical electrodes, stimuli were constructed by fixing the relative across-electrode level differences constant in dB according to the MCL profile for an 80-pps pulse train measured previously. As a way of checking whether MCL profiles measured for a low-rate (80-pps) pulse train can effectively be extrapolated to high-rate (981-pps) stimulation, an MCL profile was also measured for single-electrode stimulation with a 981-pps pulse train at the four most apical electrodes by means of loudness scaling.

Following MCL determination, rate-pitch ranking was assessed using the MPC procedure with the best-of-three adaptation as described previously. The MPC procedure was performed five times for each stimulation condition, and results were averaged across runs for each condition. Due to time constraints, rate-pitch ranking tasks for single-electrode mid-array and multi-electrode apical stimulation conditions had to be presented in separate sessions. In order to exclude potential attention and learning effects across sessions, MCLs and rate-pitch ranking for selective apical stimulation were evaluated in both sessions, and only those conditions presented within the same session were compared to each other. Within sessions, individual runs for each condition were alternately presented. The rate-pitch ranking data were analyzed in two ways. First, a broken-stick function was fitted to the rate-pitch-rank function using the matlab curve Fitting Toolbox with the same parameters used in Carlyon *et al.* (2019). The upper limit of temporal pitch perception was defined as the pulse rate corresponding to the intersection between two straight lines. The constraints were [1, 3] and [ –0.1, 0] for the slopes of the first line spanning the lower rates and the second line spanning a range of higher rates, respectively. Second, the slope of the pitch-rank function for rates equal and above 335 pps (i.e., closest to the typical upper limit of 300 pps) was calculated (Macherey *et al.*, 2011).

Statistical analyses were run using IBM SPSS Statistics 28.0.0 (IBM Corp., Armonk, NY). The log-transformed upper limits and slopes above 300 pps of the rate-pitch ranking functions were compared across stimulation conditions using paired-samples *t*-tests. In addition, effect sizes and their confidence intervals were calculated using Cohen's *d*. Finally, Pearson's correlation coefficients *r* were calculated to assess the relationship between the PE and the log-transformed upper limit.

## III. RESULTS

### A. Impedances and induced voltage recordings

Based on the line plots and heatmaps from SCINSEV recordings, two experienced senior audiologists independently estimated the number of extracochlear electrodes. Overall, their ratings were consistent, and only two cases (M014 and M035), for which estimations differed by one extracochlear electrode, had to be discussed. Their findings are presented in Table I. Only two out of eight participants had SCINSEV recordings consistent with a complete insertion of the electrode array. For the remaining six participants, SCINSEV recordings suggested incomplete insertions, with the estimated number of extracochlear electrodes ranging between 1 and 4. These estimations generally corresponded well with the number of deactivated electrodes in participants' clinical MAPs. It is unclear whether the presence of extracochlear electrodes was the result of incomplete insertion during surgery or post-implantation extrusion of the electrode array. The estimated maximum insertion depths of the most apical electrode are presented in Table I and vary between 23.1 and 30.4 mm.

### B. Polarity effect

**FIG. 3.**
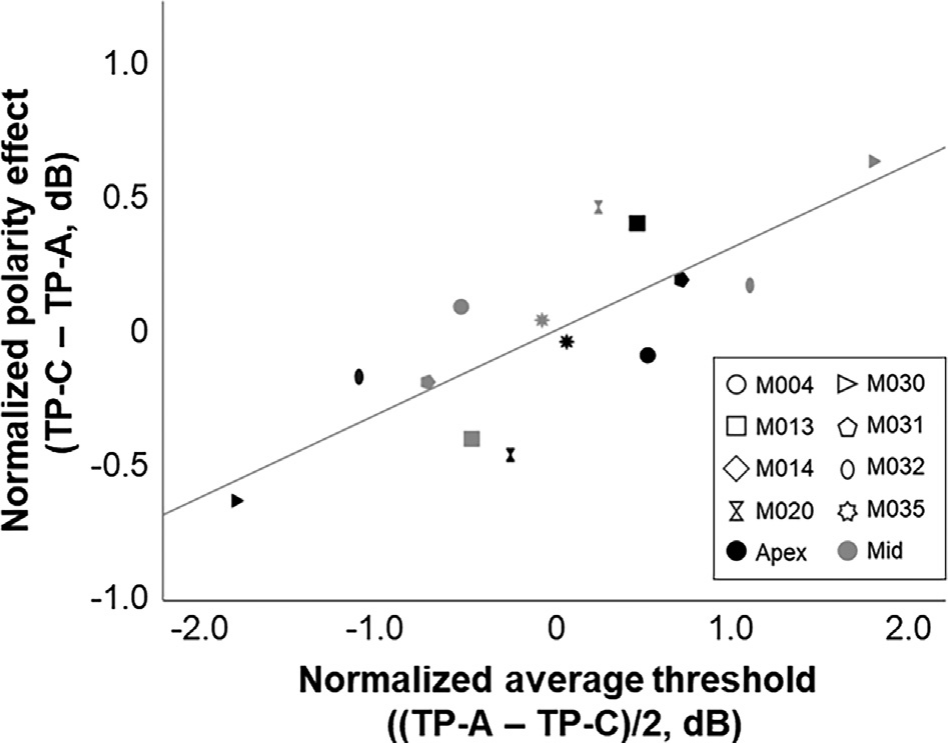
Correlation between the normalized average threshold and the normalized difference between TP-C and TP-A pulse trains (i.e., polarity effect), in dB. Data for individual participants are shown using different shapes. Black and gray symbols indicate thresholds measured for apical and mid-array stimulation, respectively.

The polarity effect was defined as the difference between TP-A and TP-C thresholds (TP-C–TP-A), which were measured between two and four times. The average *SD* for TP-A and TP-C thresholds across runs were 0.45 and 0.42 dB for the most apical electrode and 0.41 and 0.26 dB for the mid-array electrode, respectively. The correlation between the normalized PE and the average threshold [(TP-A+TP-C)/2] is shown in Fig. 3. Pearson's correlation revealed a strong and significant positive correlation between the average threshold and the PE (*r* = 0.791, *p* < 0.01, 95% CI [0.09, 0.97]), consistent with previous reports, the interpretation of which is considered further in the Sec. IV.

**FIG. 4.**
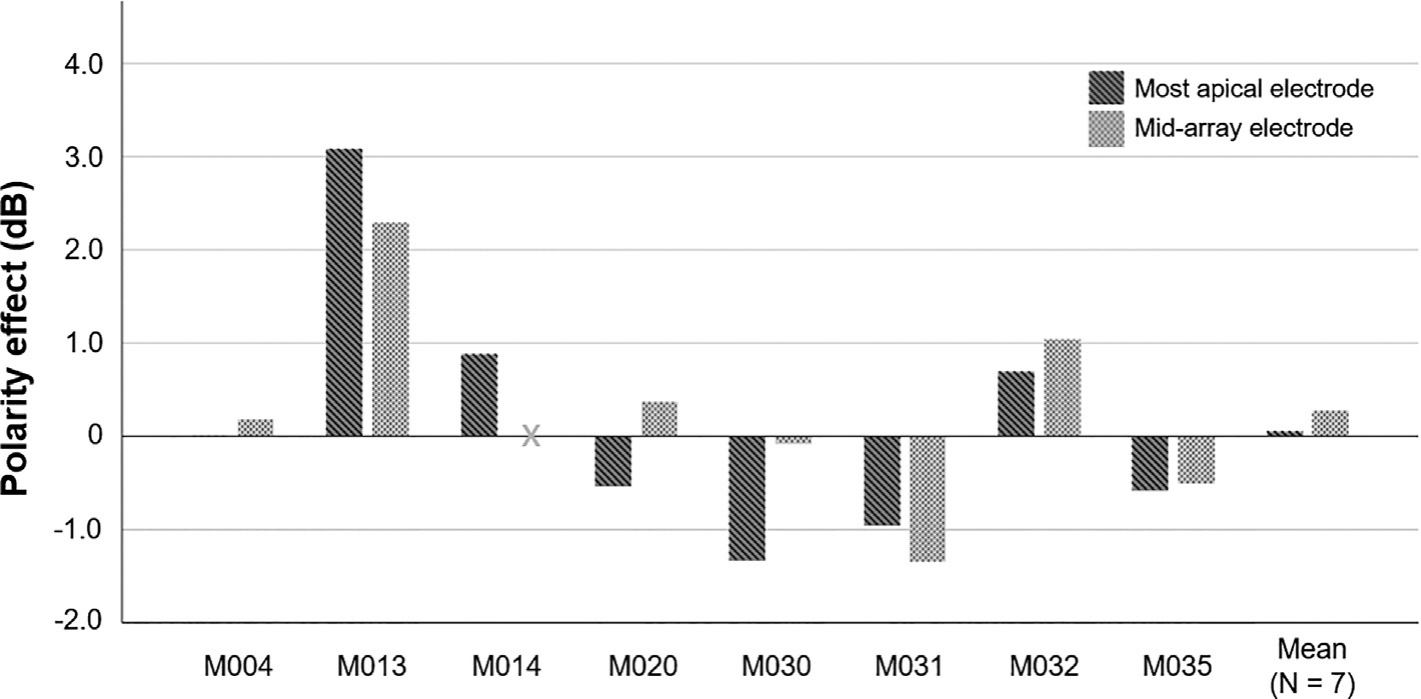
Polarity effect (PE, TP-C–TP-A) for individual participants and averaged across seven participants for whom the PE was measured at two electrodes (far right), in dB. Dark and light gray bars show the PE measured at the most apical and a mid-array electrode, respectively. The 0-dB baseline represents no difference between thresholds measured for TP-C and TP-A pulse trains. Negative values indicate good estimated neural health.

The PE (TP-C–TP-A) for individual participants and the average PE for both electrode locations are shown in Fig. 4. The PE varied considerably across participants and electrodes. As a group, the PE measured at the most apical electrode did not differ significantly from the PE measured at a mid-array electrode [*t*(6) = −0.87, *p* = 0.42], and the effect size was small (Cohen's *d* = –0.33, 95% CI [ –1.08, 0.45]).

### C. Place-pitch ranking

**FIG. 5.**
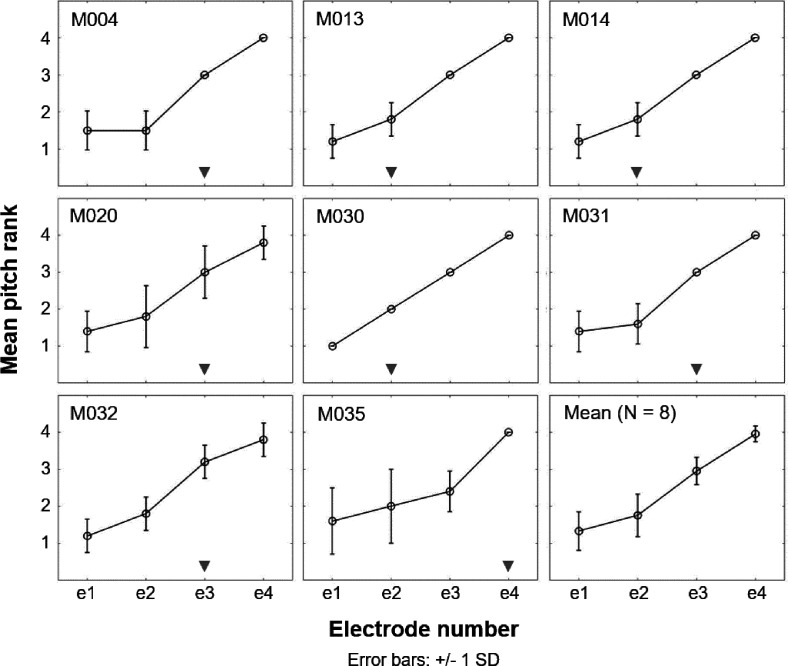
Mean place-pitch ranks and *SDs* obtained from five runs (or ten for participant M004) of the midpoint comparison procedure for individual participants and averaged across participants (bottom right). For each participant, the gray triangle at the bottom of each graph indicates the lowest-numbered electrode that produced a mean pitch rank 1 *SD* above the mean rank for e1.

The mean place-pitch ranks and *SD* for individual participants and the average pitch ranks across participants are shown in Fig. 5. In general, a pattern of monotonically increasing pitch ranks with increasing electrode numbers can be observed for most participants. However, five out of eight participants (M004, M020, M031, M032, and M035) seemed unable to discriminate e1 from e2, suggesting apical place-pitch confusion. For these participants, e3 (and e4 for participant M035) was the closest electrode that produced a pitch rank 1 *SD* above the mean rank for e1. No pitch reversals were observed. For all participants, e1 was selected as the most apical electrode in the single-electrode apical stimulation condition.

### D. Rate-pitch ranking

#### 1. Single-electrode apical vs mid-array stimulation

**FIG. 6.**
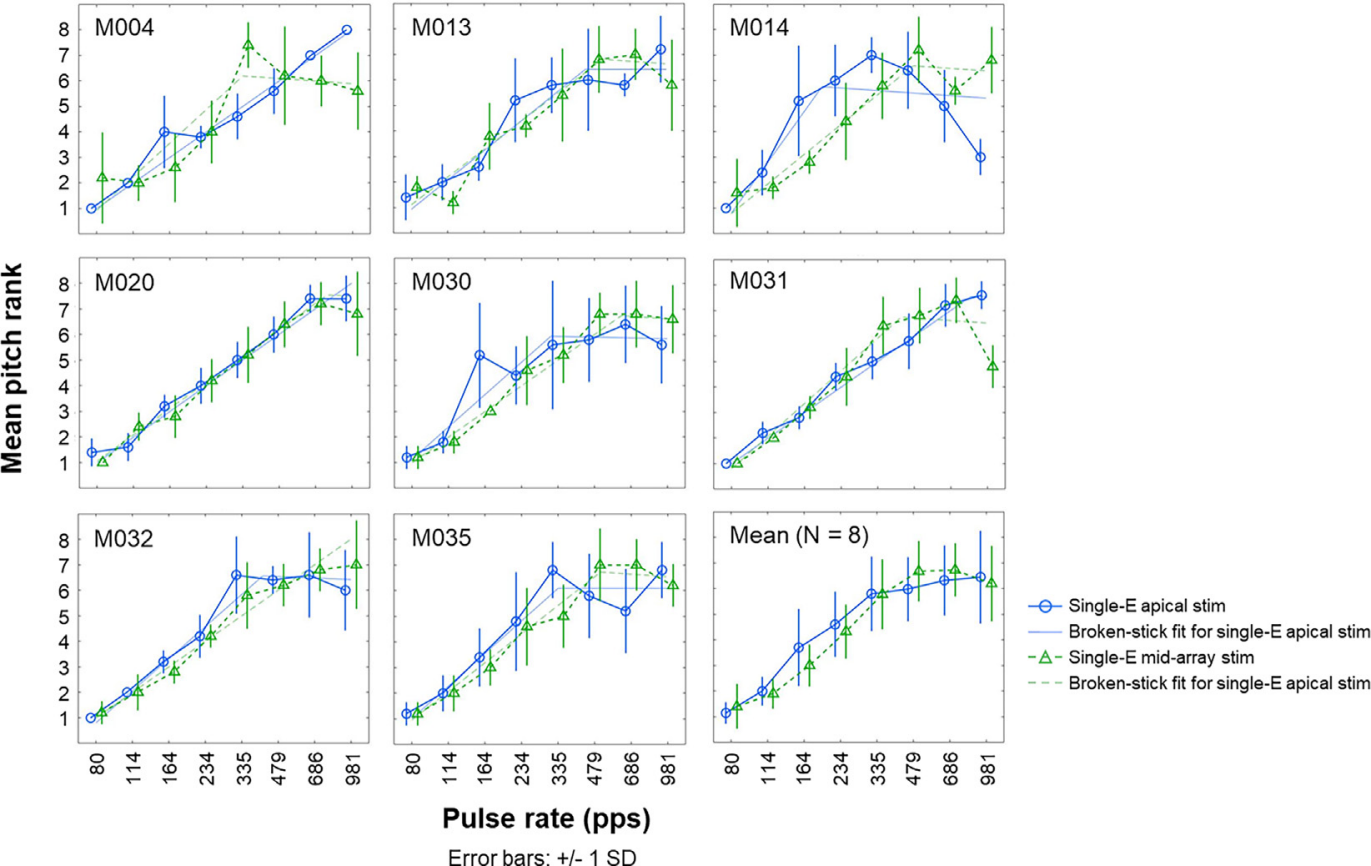
(Color online) Mean rate-pitch ranks and *SD* obtained from five runs of the midpoint comparison procedure for individual participants and averaged across participants (bottom right). Full and dashed lines with round and triangle markers show the single-electrode apical (Single-E apical stim) and mid-array (Single-E mid-array stim) stimulation conditions, respectively. Full and dashed faded lines show the broken-stick functions fitted to the data for the single-electrode apical and mid-array stimulation conditions, respectively. Upper limit values presented in Table II are defined as the pulse rate corresponding to the intersection between the two straight lines that make up the broken-fit function.

**FIG. 7.**
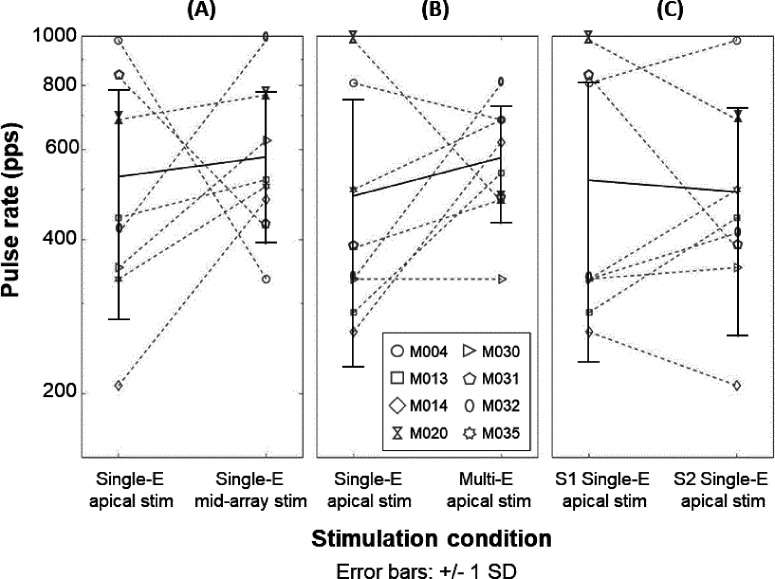
Upper limit of temporal pitch perception for individual participants (dashed gray lines) and the mean and *SD*s across participants (black). (A) shows the comparison between single-electrode apical and mid-array stimulation. (B) shows the comparison between single-electrode and multi-electrode apical stimulation. Note that rate-pitch ranking for single-electrode apical stimulation was measured on both sessions, and only those conditions measured during the same session are compared. The comparison between single-electrode apical stimulation in session 1 (S1) and session 2 (S2) is shown in (C). Table II reports the upper limit values for individual participants presented here.

The mean rate-pitch ranks and *SD* for individual participants and the average pitch ranks across participants are shown in Fig. 6. As expected, a pattern of monotonically increasing pitch ranks with increasing pulse rate and flattening of the pitch-rank function at the highest pulse rates can be observed in most participants. However, some participants showed temporal pitch reversals as reflected by decreasing pitch-rank functions at the highest pulse rates in one or more stimulation conditions (e.g., single-electrode apical stimulation for participant M014). This observation, which has been observed in several previous studies (e.g., Cosentino *et al.*, 2016), is shown in Table II by negative slopes for rates above 300 pps. Figure 7(A) shows the upper limit of temporal pitch perception for single-electrode apical and mid-array stimulation, which was derived using the broken-stick function and is presented in faded colors in Fig. 6. The mean upper limit was 531 and 580 pps for the single-electrode apical and mid-array stimulation condition, respectively. The upper limit did not differ significantly between single-electrode apical and mid-array stimulation [*t*(7) = –0.63, *p* = 0.73], and the effect size was small (Cohen's *d* = –0.22, 95% CI [–0.92, 0.49]). Similarly, there was no significant effect of the stimulation condition on the slopes above 300 pps [*t*(7) = −0.34, *p* = 0.74], and the effect size was small (Cohen's *d* = –0.12, 95% CI [–0.81, 0.58]). Furthermore, Pearson's correlation revealed no significant correlation between the PE difference measured at the most apical and a mid-array electrode on the one hand and the upper limit difference measured at those two sites on the other hand (*r* = –0.34, *p* = 0.46, 95% CI [–0.27, 0.81]).

**TABLE II.** Upper limit of temporal pitch perception and slope of the rate pitch-rank function above 300 pulses per second (pps) presented for the three comparisons. *Note.* The slope values of the rate pitch-rank function represent the change in log-transformed rate necessary for one unit increase in rate-pitch rank. *R*^2^ represents variance explained by the fitted slopes.

Experiment	Analysis	Condition	M004	M013	M014	M020	M030	M031	M032	M035
*(i) Single-electrode apical vs mid-array stimulation*	Slope (R^2^)	Single apex	0.13 (1.0)	0.23 (0.59)	−0.11 (0.95)	0.15 (0.80)	0.11 (0.04)	0.16 (0.96)	−0.51 (0.53)	−0.03 (0.01)
		Single mid	−0.24 (0.87)	0.06 (0.06)	0.06 (0.05)	0.19 (0.70)	0.18 (0.49)	−0.09 (0.24)	0.18 (0.99)	0.10 (0.24)
	Upper limit (pps)	Single apex	981	441	207	686	353	833	412	335
		Single mid	335	524	479	767	625	422	981	509
*(ii) Single-electrode vs multi-electrode apical stimulation*	Slope (R^2^)	Single apex	0.16 (0.96)	−0.12 (0.74)	0.33 (0.38)	0.17 (0.99)	−0.22 (0.75)	0.17 (0.07)	−0.18 (0.54)	0.50 (0.89)
		Multi apex	−0.05 (0.03)	0.16 (0.79)	0.23 (0.36)	1.17 (0.90)	−0.09 (0.05)	0.02 (0.00)	0.31 (0.60)	0.12 (0.70)
	Upper limit (pps)	Single apex	809	288	264	980	335	385	335	501
		Multi apex	686	540	620	475	335	479	807	686
*(iii) Single-electrode apical stimulation: Session 1 vs Session 2*	Slope (R^2^)	Session 1	0.16 (0.96)	−0.12 (0.74)	0.33 (0.38)	0.17 (0.99)	−0.22 (0.75)	0.16 (0.96)	−0.18 (0.54)	−0.03 (0.01)
		Session 2	0.13 (1.0)	0.23 (0.59)	−0.11 (0.95)	0.15 (0.80)	0.11 (0.04)	0.17 (0.07)	−0.51 (0.53)	0.50 (0.89)
	Upper limit (pps)	Session 1	809	288	264	980	335	833	335	335
		Session 2	981	441	207	686	353	385	412	501

#### 2. Single-electrode vs multi-electrode apical stimulation

**FIG. 8.**
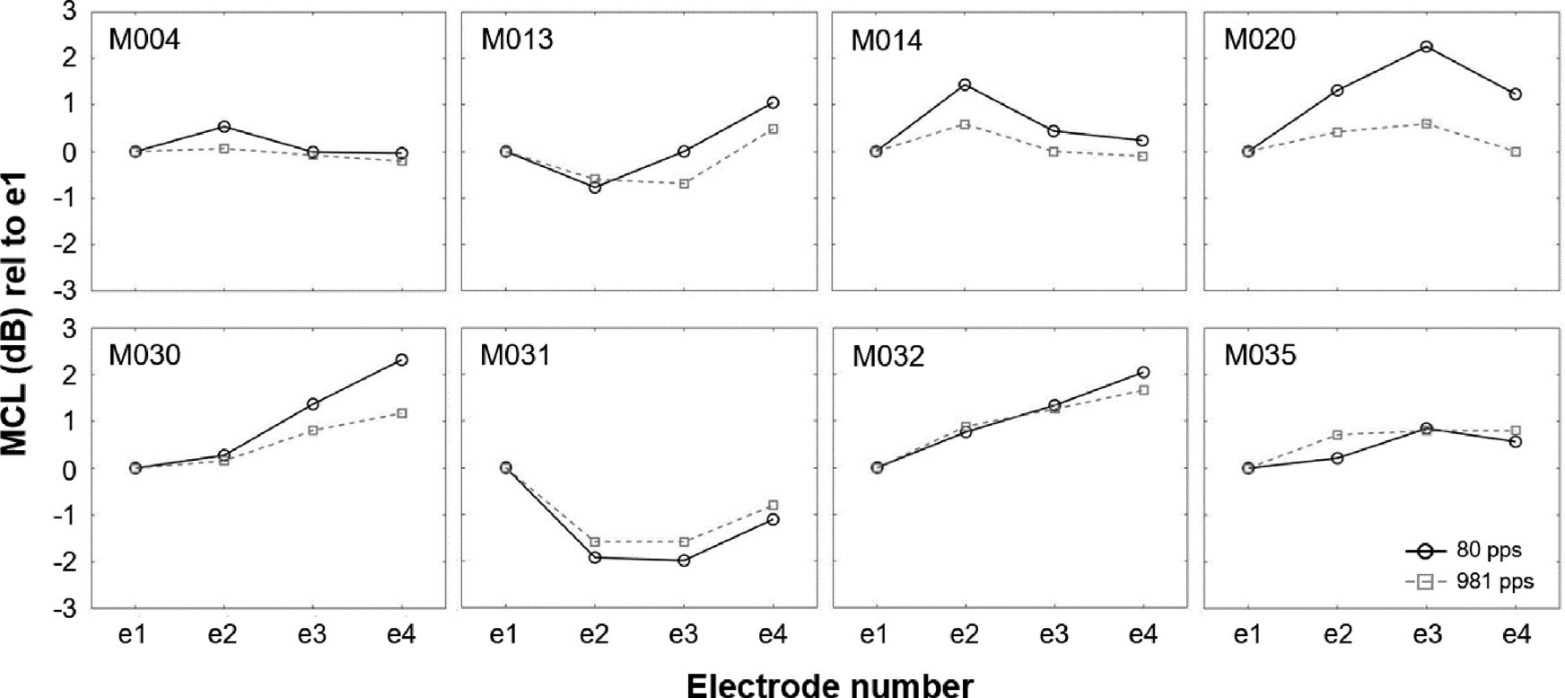
Most comfortable level (MCL) profiles for 80- and 981-pps pulse trains for individual participants. MCL profiles are shown relative to the MCL measured at the most apical electrode, e1. MCL profiles were measured by means of loudness scaling for single-electrode stimulation and used to fix the across-electrode differences for the simultaneous stimulation of the four most apical electrodes.

Figure 8 shows the MCL profiles measured for an 80-pps pulse train that were used for setting the relative across-electrode level differences (in dB) during the multi-electrode stimulation condition and the MCL profiles obtained for a high-rate 981-pps pulse train. Overall, across-electrode MCL differences follow a similar pattern for stimulation at 80 and 981 pps, although they seem less pronounced for the 981-pps pulse train in some participants (e.g., participant M020).

**FIG. 9.**
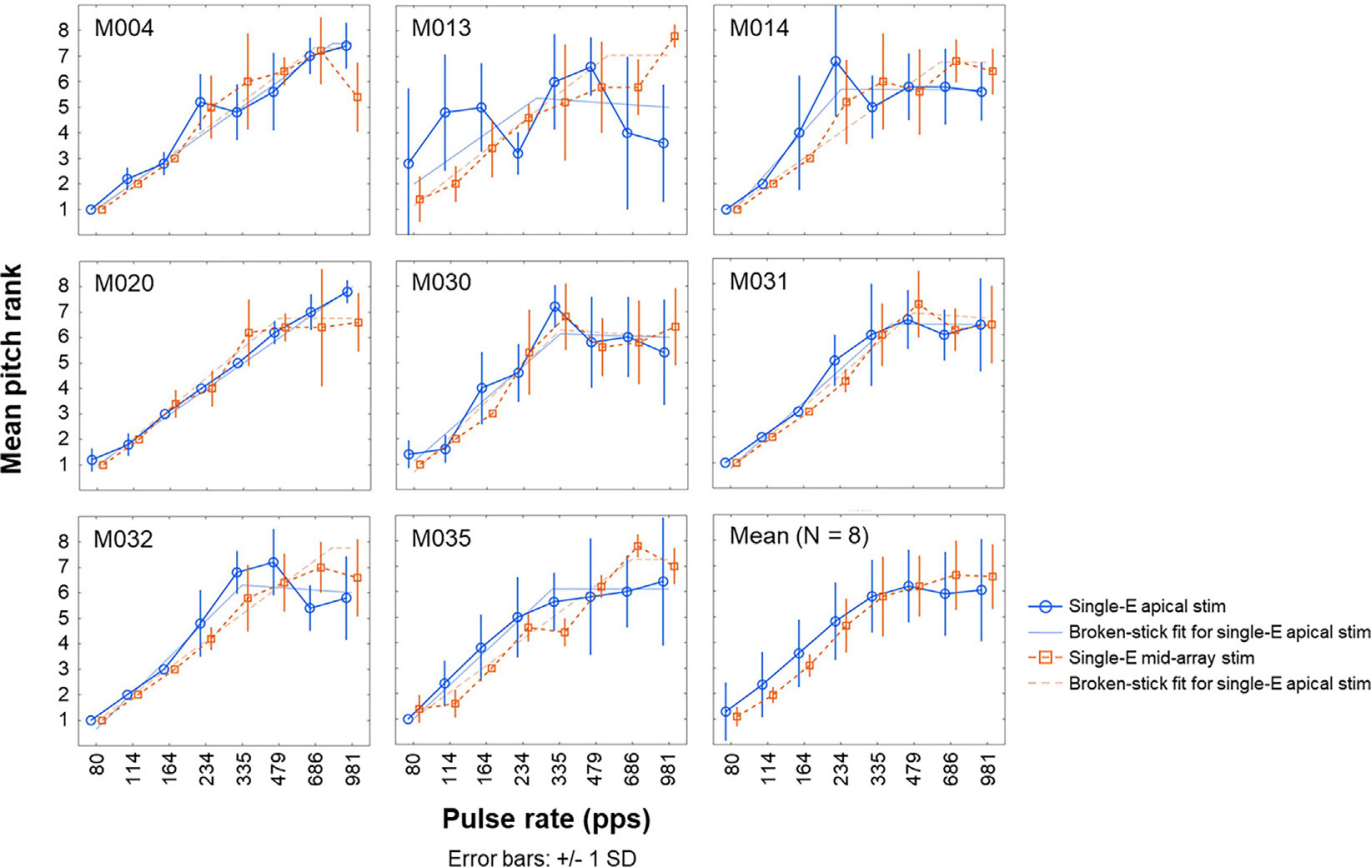
(Color online) Mean rate-pitch ranks and *SD*s obtained from five runs of the midpoint comparison procedure for individual participants and averaged across participants (bottom right). Full and dashed lines with round and square markers show the selective (Single-E apical stim) and multi-electrode apical (Multi-E apical stim) stimulation conditions, respectively. Full and dashed faded lines show the broken-stick functions fitted to the data for the single-electrode and multi-electrode apical stimulation conditions, respectively. Upper limit values presented in Table II are defined as the pulse rate corresponding to the intersection between the two straight lines that make up the broken-fit function.

The mean rate-pitch ranks and *SD* for individual participants and the average pitch ranks across participants are shown in Fig. 9. Again, most participants demonstrated a pattern of monotonically increasing pitch ranks with increasing pulse rate and flattening of the pitch-rank function at the highest pulse rates, although some participants showed decreasing pitch-rank functions at the highest pulse rates. Indeed, negative slopes above 300 pps were found for participants M013, M030, and M032 (Table II), suggesting temporal pitch reversals. The upper limit of temporal pitch perception for the single-electrode and multi-electrode apical stimulation condition is shown in Fig. 7B. The mean upper limit was 487 and 579 pps for the single-electrode and multi-electrode apical stimulation condition, respectively. Neither the upper limit [*t*(7) = –1.31, *p* = 0.23], nor the slope above 300 pps [*t*(7) = –0.83, *p* = 0.43] differed significantly between stimulation conditions. Small effect sizes were found for both the upper limit (Cohen's *d* = –0.46, 95% CI [–1.18, 0.29]) and slope values (Cohen's *d* = –0.30, 95% CI [–0.99, 0.43]). Furthermore, the correlation between the PE measured at the apex on the one hand and the upper limit difference measured for single-electrode and multi-electrode apical stimulation on the other hand was not significant (*r* = –0.55, *p* = 0.16, 95% CI [ –0.97, –0.17]).

#### 3. Single-electrode apical stimulation: Session 1 vs session 2

Rate-pitch ranking for single-electrode apical stimulation was repeated on each session. This allowed us to compare between-sessions estimates of temporal pitch sensitivity, shown in Fig. 7(C). The mean upper limit was 522 and 496 pps for single-electrode apical stimulation measured during the first and second session, respectively. As a group, the upper limit did not differ significantly in the first session compared to the second session [*t*(7) = 0.10, *p* = 0.92], nor did the slope above 300 pps [*t*(7) = −0.41, *p* = 0.70]. Effect sizes were small for both the upper limit (Cohen's *d* = 0.04, 95% CI [–0.66, 0.73]) and slope values (Cohen's *d* = –0.14, 95% CI [–0.84, 0.56]). However, as can be observed in Table II and by comparing the full blue traces in Figs. 6–9, some participants (e.g., participant M031) seemed to demonstrate relatively non-stable rate-pitch ranking for the single-electrode apical stimulation across sessions. Across participants, Pearson's correlation suggested a strong correlation between the upper limit measured during session 1 and session 2, but this did not reach statistical significance (*r* = 0.67, *p* = 0.07, 95% CI [–0.01, 0.95]).

## IV. DISCUSSION

The primary aim of this study was to investigate whether selective apical stimulation can improve temporal pitch processing in human CI recipients and whether any such improvement relates to the survival of the peripheral processes of the AN. To this end, we used a rate-pitch ranking task and compared the upper limit of temporal pitch perception and the slope of the pitch-rank function above 300 pps for (i) single-electrode apical stimulation, (ii) stimulation of a single mid-array electrode, and (iii) simultaneous multi-electrode apical stimulation. Our results did not show a significant improvement of single-electrode apical stimulation on temporal pitch processing compared to other stimulation conditions. As part of our investigation, we also obtained estimates of the number of extracochlear electrodes to gauge insertion depth and of the survival of peripheral processes using the PE, together with a measure of the selectivity of apical stimulation using a place-pitch ranking task. This section starts with a consideration of these measures before discussing their implications on the temporal pitch results.

### A. Extracochlear electrodes

As can be seen in Table I, six out of eight participants (75%) demonstrated SCINSEV recordings consistent with at least one and up to four extracochlear electrodes. This number is considerably higher than the prevalence reported in a retrospective study by Holder *et al.* (2018), who detected extracochlear electrodes in 13.4% of CI recipients. However, in the same study, the prevalence of extracochlear electrodes was slightly higher (26.6%) for MED-EL devices compared to other manufacturers. Reassuringly, the number of extracochlear electrodes identified here generally corresponded well (± 1) with the number of deactivated electrodes in participants' clinical MAP, with the exception of participant M004. Although the presence of extracochlear electrodes can provide some information about the insertion depth, other techniques are required to inform about the insertion angle, which is of importance given the between-subject variability of cochlear duct length (Hardy, 1938). Nevertheless, as the benefit of selective apical stimulation necessarily relies on a deep insertion of the electrode array into the apex of the cochlea, the results of this study should be interpreted with the knowledge that we may not have been stimulating sufficiently apically to observe a measurable improvement of temporal pitch perception with selective stimulation of the apical electrodes.

### B. Polarity effect

The data shown in Fig. 3 demonstrate that the PE correlates positively with the mean of the detection thresholds obtained with TP-A and TP-C pulses, consistent with previous findings. Inspection of Fig. 3 suggests that participant M030 contributed most to this correlation, but we note that the rank-order coefficient was also statistically significant (Spearman's rho = 0.754, *p* < 0.01), suggesting that the correlation was not overly influenced by this outlier. The confidence intervals were necessarily wide, due to the modest number of participants, but the finding adds to a body of evidence from a number of studies that have also reported that the PE correlates, across electrodes, significantly with the detection thresholds for a train of symmetric pulses or with the mean of the detection thresholds obtained with anodic-dominant and cathodic-dominant asymmetric pulses (Brochier *et al.*, 2021; Carlyon *et al.*, 2018; Jahn and Arenberg 2019a,b; Mesnildrey *et al.*, 2020). The authors of those studies proposed a simple explanation, which is that the thresholds overall depend on both the distance of the electrodes from the auditory neurons and on the survival of the peripheral processes, and that peripheral-process survival affects the PE. A different explanation was recently proposed in a computational study by Kalkman *et al.* (2022), who simulated thresholds for a range of different cochlear geometries, array types, and insertion depths of the simulated electrode. They were able to replicate the correlation shown by Carlyon *et al.* (2018) and replicated here using neurons with uniformly perfect survival of the peripheral processes and concluded that the PE is not a reliable measure of peripheral-process survival. However, the correlation reported by Kalkman *et al.* (2022) was entirely accounted for by insertion depth, with more deeply-inserted electrodes showing more negative PEs (i.e., lower thresholds for cathodic-dominant than for anodic-dominant pulses). In contrast to this prediction, as they point out, previous studies using Cochlear and Advanced Bionics devices have not observed a consistent decrease in the PE with more-apical compared to mid-array or more-basal electrodes. For example, Brochier *et al.* (2021) reported highly significant correlations between the PE and both the average threshold obtained with anodic-dominant and cathodic-dominant pulses and with a symmetric biphasic pulse. We have re-analyzed their data so as to calculate the correlation between electrode number and the PE while removing between-participant differences; the resulting correlation coefficient was 0.002. The discrepancy between the Kalkman *et al.* model predictions and experimental data is perhaps even clearer here, where we compared thresholds and the PE for e1 vs e7 or e8 of the MED-EL device, and where array lengths were always 28 or 31.5 mm (with electrode spacings of 2.4 and 2.1 mm, respectively). Although we do not have post-operative CT scans for our participants, the insertion angle for these two electrodes must still differ greatly. For complete insertions, Landsberger *et al.* (2015) reported average insertion angles of 174 and 544 degrees for electrodes 7 and 1 of the MED-EL standard 31.5-mm array, respectively. For shorter 28-mm arrays, insertion angles were lower on average, at 174 and 471 degrees for electrodes 7 and 1, respectively. Despite this huge difference in insertion depth, the PE measured in this study and presented in Fig. 4 did not differ consistently between apical and mid-array electrodes. Hence, there remains no evidence that it is possible to account for the observed across-electrode variations in PE without the recourse to the survival of the peripheral AN processes.

### C. Place pitch

Place-pitch encoding of the four most apical electrodes was evaluated to verify the pitch and discriminability of e1, as previous studies have shown apical place-pitch encoding to be especially poor in MED-EL CI recipients (Baumann and Nobbe, 2006; Boyd, 2011; Gani *et al.*, 2007; Kenway *et al.*, 2015). We expected participants with accurate apical place-pitch perception to benefit most from selective apical stimulation, as selective stimulation is a prerequisite for distinguishing stimulation at different electrodes. Consistent with earlier studies, the data presented in Fig. 5 show place-pitch confusions of the two most apical electrodes in more than half of the participants; although none showed true place-pitch reversals such as have been reported in some previous studies. In contrast, e3 and e4 could generally be accurately discriminated. This finding suggests that the two most apical electrodes may have been stimulating largely overlapping neural populations, which will likely have limited the selectivity of the single-electrode apical stimulation condition used here. Selective electrical stimulation of the cochlear apex is hampered by the fact that spiral ganglion cells do not extend all the way into the apex, and that the low-frequency AN fibers are innervated by peripheral processes originating from tightly packed cell bodies at more basal sites (Ariyasu *et al.*, 1989; Spoendlin and Schrott, 1989; Stakhovskaya *et al.*, 2007). For this reason, it is generally assumed that good peripheral neural health is a prerequisite for selective electrical stimulation at the apex (Croner *et al.*, 2022; Briaire and Frijns, 2006), and hence, accurate place-pitch encoding (Baumann and Nobbe, 2006; Deman *et al.*, 2004; Landsberger *et al.*, 2014). In contrast, the status of peripheral AN processes may have less of an impact on place-pitch encoding at the basal end of the cochlea, as basic cochlear anatomy predicts that electrical stimulation will excite the AN fibers in the vicinity of the electrode, irrespective of the site of spike initiation. This may explain why, based on an informal analysis of their data, Baumann and Nobbe (2006) noted that place-pitch confusions of the two most apical electrodes occurred mostly for participants with “comparably deeper insertions of the electrode.” Inspection of Table I of the present study shows that participant M035, who showed extensive apical place-pitch confusions, had a fully inserted 31.5-mm electrode array and the highest estimated maximum insertion depth—consistent with Baumann and Nobbe (2006). However, participant M020, who has four extracochlear electrodes and the lowest estimated maximum insertion depth, also demonstrates place-pitch confusion. In between these two extremes, estimated maximum insertion depths vary between 23.1 and 27.1 mm, and no clear pattern of apical place-pitch encoding emerges. A caveat is that the insertion depths presented here are merely an estimate based on the number of extracochlear electrodes and assuming a “best-case scenario” for all participants.

Our estimate of local neural health presented on Fig. 4, the PE, suggested generally poor peripheral neural survival at the most apical electrode in this group of participants, with only four out of eight participants demonstrating negative PE values (i.e., better estimated neural health). However, we did not observe the predicted relationship between the PE and place-pitch encoding at the most apical electrodes. For example, participants M013, M014, and M032 showed positive PE values (i.e., poorer predicted neural health) and good apical place-pitch encoding, whereas for participants M020, M031, and M035, negative PE (i.e., better predicted neural health) values were accompanied by poor apical place-pitch encoding. One possible reason why the PE did not predict place-pitch encoding is that, although peripheral-process survival may be necessary for good place-pitch perception at the apex, it may not be sufficient. For example, Kalkman *et al.* (2014) modelled the course of peripheral processes and showed that electrodes in the cochlear apex can excite peripheral processes that project obliquely from more-apical cochlear locations, leading to predicted distortions in the representation of place pitch. Furthermore, six out of eight participants were judged to have at least one and up to four extracochlear electrodes, which may have limited the most apical nerve fibers that could be stimulated. Hence, in some participants, e1 and e2 may not have been inserted beyond the end of the spiral ganglion, where degeneration of the peripheral AN processes is expected to affect place-pitch encoding. With the most apical electrodes not being inserted sufficiently deep into the cochlea, either because participants were implanted with the shorter 28-mm array or had incomplete insertions, the relationship between peripheral neural health and place-pitch encoding may have been obscured, and accurate place-pitch encoding of the most apical electrodes could be accompanied by poor neural health in the same cochlear region.

### D. Temporal pitch

As expected, the data shown in Figs. 6 and 9 demonstrate that pitch was rated as increasingly higher with increasing pulse rates for all stimulation conditions, up until about 500 pps on average, consistent with previous studies that employed similar rate-pitch ranking tasks (e.g., Carlyon *et al.*, 2010). Overall, the upper limit was highly variable across participants, with some participants (e.g., M020) performing exceptionally well and seemingly able to reliably discriminate rates higher than 500 pps (Kong and Carlyon, 2010). Other participants showed temporal pitch reversals, in which one or more high-rate pulse trains evoked lower pitch ranks than lower pulse rates (e.g., M014). These temporal pitch reversals were only observed at rates above participants' upper limit of temporal pitch perception. Temporal pitch reversals have been observed at high rates in previous studies, but their nature remains unclear at present (Kong and Carlyon 2010; Lamping *et al.*, 2020; Macherey *et al.*, 2011; Cosentino *et al.*, 2016).

With single-electrode stimulation of the most apical electrode, we attempted to selectively stimulate the AN fibers with low characteristic frequencies in order to target a specialized low-frequency brainstem pathway that has been proposed to support precise temporal processing (Middlebrooks and Snyder, 2010). However, contrary to our expectations, single-electrode apical stimulation did not result in superior temporal pitch processing when compared to a stimulation condition in which the place of excitation was shifted toward more basal cochlear sites. This lack of a benefit did not seem to be restricted to participants showing poor spatial selectivity at the apex. For example, participant M030 showed good estimated peripheral neural survival at the apex, but not at the mid-array electrode. As expected, they demonstrated good place-pitch encoding at the apex of the cochlea but showed rate-pitch ranking for single-electrode apical stimulation that was worse than for a mid-array electrode and no better than for multi-electrode apical stimulation conditions. Moreover, in this group of CI recipients, effectively reducing the selectivity at the apex by presenting the same stimulus to the four most apical electrodes simultaneously, did not significantly affect temporal pitch processing.

There are several possible reasons for the finding that performance with single-electrode apical stimulation did not improve temporal pitch perception relative to other stimulation conditions. First, SCINSEV recordings suggested that most participants had at least one and up to four extracochlear electrodes, necessarily limiting the insertion depth of the electrode array. For these participants, the most apical electrode may not have been inserted sufficiently deep to effectively access the low-frequency AN fibers that are thought to support more precise temporal pitch processing. As described in the following, an informal analysis of the insertion depth vs the upper limit does not point to a clear relationship between the two. On the one hand, only two out of eight participants (M004 and M031) numerically showed the expected benefit of apical compared to mid-array stimulation, and only one participant (M020) showed higher upper limits for single-electrode compared to multi-electrode apical stimulation. These participants were all implanted with the longest 31.5-mm array, but they all had at least two extracochlear electrodes, evidently limiting insertion depth. On the other hand, participant M035, who had a full insertion of the longest array and thus a good chance of being stimulated sufficiently apically (albeit not selectively based on the results from apical place-pitch encoding), showed no improvement with selective apical stimulation compared to the other two stimulation conditions.

Second, it could simply be that selective apical stimulation may not be important for temporal pitch perception. The primary evidence for a role for selective apical stimulation comes from recordings from the IC of anaesthetized cats, and it is not known whether this would lead to an improvement in perception in an awake cat or human (Middlebrooks and Snyder, 2010). Evidence for improved temporal processing at the apex has been obtained by Stahl *et al.* (2016), both in NH and CI listeners, but this was observed only for the discrimination of low pulse rates rather than the upper limit tested here. As noted in the Introduction, Lamping *et al.* (2020) and Macherey *et al.* (2011) did observe an increase in the upper limit of temporal pitch using a stimulus manipulation—a combination of bipolar stimulation and asymmetric pulse shapes—that produced a place of excitation more apical than the most apical electrode. However, they suggested that the improvement might not be due to selective apical stimulation per se, but rather to the stimulation of neural sites that had not been damaged by electrode insertion. If correct, then one would not necessarily expect better temporal pitch coding for an apical vs a mid-array electrode.

A third explanation is that selective apical stimulation is important but that, in human CI recipients, its effect is swamped by variations in neural health, either at the AN and/or more centrally, that are not captured by the PE. A recent study by Brochier *et al.* (2021) demonstrated that various neural health measures, including the PE, multi-pulse integration, and electrically-evoked compound action potential, may indeed reflect different characteristics of the electrode-neural interface.

Fourth, it is possible that the single-electrode and multi-electrode apical stimulation conditions used here were not different enough, as stimulation in both conditions was applied using a lateral-wall electrode in monopolar mode and at MCL, which inherently causes a relatively broad spread of excitation, even with single-electrode stimulation (Goldwyn *et al.*, 2010). If this is correct, then a device that allows focused stimulation combined with a long electrode array might provide the necessary selective-apical excitation.

Finally, it is possible that the stimuli that were pitch-ranked differed along another perceptual dimension, and that this somehow affected the results. With regard to residual loudness differences, we took great care to loudness balance the stimuli used in the rate-pitch-ranking task. Nevertheless, the relative levels of the four most apical electrodes that were stimulated simultaneously in the multi-electrode apical condition were the same at all rates, and comparison of the MCL profile at 80 vs 981 pps in Fig. 8 revealed considerable differences in some participants (e.g., M020 and M030). Despite generally following the same pattern of MCLs across electrodes for both rates, it seemed that the MCL profile measured at 981 pps was generally flatter than the MCL profile measured at 80 pps, indicating that between-electrode differences in MCL were less pronounced at higher rates. This may have led to a progressive overstimulation of more basal electrodes and thus an increasingly higher place-pitch cue with increasing pulse rate for multi-electrode stimulation. Note that this effect would potentially have increased the estimated upper limit for multi-electrode apical stimulation. Interestingly though, participants M020 and M030, who both showed this effect of rate on MCL profiles, either showed a lower upper limit in the multi-electrode apical condition or similar limits in the selective and multi-electrode apical stimulation conditions. Therefore, it seems unlikely that the generalization of MCL-profiles across rates can explain the absence of difference between single- and multi-electrode apical stimulation conditions.

### E. Practical applications

CIs manufactured by the Advanced Bionics and MED-EL companies incorporate signal-processing strategies that modify the conventional Continuous Interleaved Sampling method so as to convey temporal fine structure (TFS) cues. The fine-structure stimulation class of strategies incorporated in the MED-EL device does so with the express aim of improving temporal pitch perception. In both devices, the TFS cues are applied only to the most apical electrodes, and so their success rests on the ability of CI recipients to exploit those cues to perceive pitch. The encouraging aspect of our results is that, on average, pitch continued to increase with increasing pulse rate up to at least 500 pps. However, it should be noted that this does not necessarily mean that listeners perceive a pitch corresponding to 500 pps, as the function relating pulse rate to pitch may well start to flatten off below this value, as has been observed with analogous stimuli in NH listeners (Macherey and Carlyon, 2014). A more thought-provoking implication of our results is that they provide no justification for conveying TFS cues specifically or exclusively to the apical electrodes compared to other electrodes in the array. For example, a strategy that extracted the fundamental frequency and presented it explicitly on one or more electrodes might instead profit from choosing electrodes based on psychophysical performance rather than on electrode location (cf. a similar argument for lateralization cues proposed by Thakkar *et al.*, (2023).

## V. CONCLUSION

This study investigated the effect of selective apical stimulation on temporal pitch perception in CI recipients. Changing the place of stimulation or the spread of excitation had no consistent effect on rate-pitch ranking. Hence, we found no improvement of selective apical stimulation in this group of CI recipients. One reason might be that we were not stimulating sufficiently apically, as SCINSEV recordings indicated that the majority of participants had incomplete insertions of the electrode array, necessarily limiting insertion depth. Moreover, the upper limit did not correlate with another psychophysical measure, the PE, which has been proposed as an estimate of local neural health.

## Data Availability

The data and code that support the findings of this study will be made available upon request from the corresponding author.

## References

[1] Adel, Y., Nagel, S., Weissgerber, T., Baumann, U., and Macherey, O. (2019). “ Pitch matching in cochlear implant users with single-sided deafness: Effects of electrode position and acoustic stimulus type,” Front. Neurosci. 13, 1–15. 10.3389/fnins.2019.01119 31736684 PMC6839387

[2] Arenberg, J. G., Parkinson, W. S., Litvak, L., Chen, C., Kreft, H. A., and Oxenham, A. J. (2018). “ A dynamically focusing cochlear implant strategy can improve vowel identification in noise,” Ear Hear. 39(6), 1136–1145. 10.1097/AUD.0000000000000566 29529006 PMC6129442

[3] Ariyasu, L., Galey, F. R., Hilsinger, R., Jr., and Byl, F. M. (1989). “ Computer‐generated three‐dimensional reconstruction of the cochlea,” Otolaryngol. Head. Neck Surg. 100(2), 87–91. 10.1177/019459988910000201 2495514

[5] Bahmer, A., and Baumann, U. (2013). “ New parallel stimulation strategies revisited: Effect of synchronous multi electrode stimulation on rate discrimination in cochlear implant users,” Cochlear Implants Int. 14(3), 142–149. 10.1179/1754762812Y.0000000011 22733121

[6] Baumann, U., and Nobbe, A. (2004). “ Pulse rate discrimination with deeply inserted electrode arrays,” Hear. Res. 196(1–2), 49–57. 10.1016/j.heares.2004.06.008 15464301

[7] Baumann, U., and Nobbe, A. (2006). “ The cochlear implant electrode–pitch function,” Hear. Res. 213(1–2), 34–42. 10.1016/j.heares.2005.12.010 16442249

[8] Boyd, P. J. (2011). “ Potential benefits from deeply inserted cochlear implant electrodes,” Ear Hear. 32(4), 411–427. 10.1097/AUD.0b013e3182064bda 21248642

[9] Briaire, J. J., and Frijns, J. H. (2006). “ The consequences of neural degeneration regarding optimal cochlear implant position in scala tympani: A model approach,” Hear. Res. 214(1–2), 17–27. 10.1016/j.heares.2006.01.015 16520009

[10] Brochier, T., Guerit, F., Deeks, J. M., Garcia, C., Bance, M., and Carlyon, R. P. (2021). “ Evaluating and comparing behavioural and electrophysiological estimates of neural health in cochlear implant users,” J. Assoc. Res. Otolaryngol. 22, 67–80. 10.1007/s10162-020-00773-0 33150541 PMC7822986

[11] Carlyon, R. P., Cosentino, S., Deeks, J. M., Parkinson, W., and Arenberg, J. A. (2018). “ Effect of stimulus polarity on detection thresholds in cochlear implant users: Relationships with average threshold, gap detection, and rate discrimination,” J. Assoc. Res. Otolaryngol. 19, 559–567. 10.1007/s10162-018-0677-5 29881937 PMC6226408

[12] Carlyon, R. P., and Deeks, J. M. (2002). “ Limitations on rate discrimination,” J. Acoust. Soc. Am. 112(3), 1009–1025. 10.1121/1.1496766 12243150

[15] Carlyon, R. P., Deeks, J. M., and Macherey, O. (2013). “ Polarity effects on place pitch and loudness for three cochlear-implant designs and at different cochlear sites,” J. Acoust. Soc. Am. 134(1), 503–509. 10.1121/1.4807900 23862825

[16] Carlyon, R. P., Deeks, J. M., and McKay, C. M. (2010). “ The upper limit of temporal pitch for cochlear-implant listeners: Stimulus duration, conditioner pulses, and the number of electrodes stimulated,” J. Acoust. Soc. Am. 127(3), 1469–1478. 10.1121/1.3291981 20329847

[17] Carlyon, R. P., Guérit, F., Billig, A. J., Tam, Y. C., Harris, F., and Deeks, J. M. (2019). “ Effect of chronic stimulation and stimulus level on temporal processing by cochlear implant listeners,” J. Assoc. Res. Otolaryngol. 20, 169–185. 10.1007/s10162-018-00706-y 30543016 PMC6453997

[20] Cohen, J. (1992). “ A power primer,” Psychol. Bull. 112(1), 155–159. 10.1037/0033-2909.112.1.155 19565683

[21] Cosentino, S., Carlyon, R. P., Deeks, J. M., Parkinson, W., and Bierer, J. A. (2016). “ Rate discrimination, gap detection and ranking of temporal pitch in cochlear implant users,” J. Assoc. Res. Otolaryngol. 17, 371–382. 10.1007/s10162-016-0569-5 27101997 PMC4940289

[22] Croner, A. M., Heshmat, A., Schrott-Fischer, A., Glueckert, R., Hemmert, W., and Bai, S. (2022). “ Effects of degrees of degeneration on the electrical excitation of human spiral ganglion neurons based on a high-resolution computer model,” Front. Neurosci. 16, 914876. 10.3389/fnins.2022.914876 35873813 PMC9298973

[23] Deman, P. R., van Dijk, B., Offeciers, F. E., and Govaerts, P. J. (2004). “ Pitch estimation of a deeply inserted cochlear implant electrode,” Int. J. Audiol. 43(6), 363–368. 10.1080/14992020400050046 15457819

[24] de Rijk, S. R., Tam, Y. C., Carlyon, R. P., and Bance, M. L. (2020). “ Detection of extracochlear electrodes in cochlear implants with electric field imaging/transimpedance measurements: A human cadaver study,” Ear Hear. 41(5), 1196–1207. 10.1097/AUD.0000000000000837 31923041 PMC7115972

[25] Dhanasingh, A., and Hochmair, I. (2021). “ Signal processing & audio processors,” Acta Otolaryngol. 141, 106–134. 10.1080/00016489.2021.1888504 33818264

[26] Gani, M., Valentini, G., Sigrist, A., Kós, M. I., and Boëx, C. (2007). “ Implications of deep electrode insertion on cochlear implant fitting,” J. Assoc. Res. Otolaryngol. 8, 69–83. 10.1007/s10162-006-0065-4 17216585 PMC2538415

[28] Goehring, T., Arenberg, J. G., and Carlyon, R. P. (2020). “ Using spectral blurring to assess effects of channel interaction on speech-in-noise perception with cochlear implants,” J. Assoc. Res. Otolaryngol. 21(4), 353–371. 10.1007/s10162-020-00758-z 32519088 PMC7445227

[29] Goldsworthy, R. L., and Shannon, R. V. (2014). “ Training improves cochlear implant rate discrimination on a psychophysical task,” J. Acoust. Soc. Am. 135(1), 334–341. 10.1121/1.4835735 24437773 PMC3985914

[30] Goldwyn, J. H., Bierer, S. M., and Bierer, J. A. (2010). “ Modeling the electrode–neuron interface of cochlear implants: Effects of neural survival, electrode placement, and the partial tripolar configuration,” Hear. Res. 268(1–2), 93–104. 10.1016/j.heares.2010.05.005 20580801 PMC2923246

[31] Hardy, M. (1938). “ The length of the organ of Corti in man,” Am. J. Anat. 62(2), 291–311. 10.1002/aja.1000620204

[32] Holder, J. T., Kessler, D. M., Noble, J. H., Gifford, R. H., and Labadie, R. F. (2018). “ Prevalence of extracochlear electrodes: Computerized tomography scans, cochlear implant maps, and operative reports,” Otol. Neurotol. 39(5), e325–e331. 10.1097/MAO.0000000000001818 29738386 PMC7197293

[33] Ihlefeld, A., Carlyon, R. P., Kan, A., Churchill, T. H., and Litovsky, R. Y. (2015). “ Limitations on monaural and binaural temporal processing in bilateral cochlear implant listeners,” J. Assoc. Res. Otolaryngol. 16, 641–652. 10.1007/s10162-015-0527-7 26105749 PMC4569611

[34] Jahn, K. N., and Arenberg, J. G. (2019a). “ Evaluating psychophysical polarity sensitivity as an indirect estimate of neural status in cochlear implant listeners,” J. Assoc. Res. Otolaryngol. 20(4), 415–430. 10.1007/s10162-019-00718-2 30949879 PMC6646612

[35] Jahn, K. N., and Arenberg, J. G. (2019b). “ Polarity sensitivity in pediatric and adult cochlear implant listeners,” Trends Hear. 23, 1–22. 10.1177/2331216519862987 PMC6681263 31373266

[36] Kalkman, R. K., Briaire, J. J., Dekker, D. M., and Frijns, J. H. (2014). “ Place pitch versus electrode location in a realistic computational model of the implanted human cochlea,” Hear. Res. 315, 10–24. 10.1016/j.heares.2014.06.003 24975087

[37] Kalkman, R. K., Briaire, J. J., Dekker, D. M., and Frijns, J. H. (2022). “ The relation between polarity sensitivity and neural degeneration in a computational model of cochlear implant stimulation,” Hear. Res. 415, 108413. 10.1016/j.heares.2021.108413 34952734

[38] Kenway, B., Tam, Y. C., Vanat, Z., Harris, F., Gray, R., Birchall, J., Carlyon, R., and Axon, P. (2015). “ Pitch discrimination: An independent factor in cochlear implant performance outcomes,” Otol. Neurotol. 36(9), 1472–1479. 10.1097/MAO.0000000000000845 26375968 PMC4588601

[39] Kong, Y. Y., and Carlyon, R. P. (2010). “ Temporal pitch perception at high rates in cochlear implants,” J. Acoust. Soc. Am. 127(5), 3114–3123. 10.1121/1.3372713 21117760

[40] Kong, Y. Y., Deeks, J. M., Axon, P. R., and Carlyon, R. P. (2009). “ Limits of temporal pitch in cochlear implants,” J. Acoust. Soc. Am. 125(3), 1649–1657. 10.1121/1.3068457 19275322

[42] Lamping, W., Deeks, J. M., Marozeau, J., and Carlyon, R. P. (2020). “ The effect of phantom stimulation and pseudomonophasic pulse shapes on pitch perception by cochlear implant listeners,” J. Assoc. Res. Otolaryngol. 21, 511–526. 10.1007/s10162-020-00768-x 32804337 PMC7644600

[43] Landsberger, D. M., Mertens, G., Punte, A. K., and Van De Heyning, P. (2014). “ Perceptual changes in place of stimulation with long cochlear implant electrode arrays,” J. Acoust. Soc. Am. 135(2), EL75–EL81. 10.1121/1.4862875 25234918 PMC3985910

[44] Landsberger, D. M., Svrakic, S., Roland, J. T., and Svirsky, M. (2015). “ The relationship between insertion angles, default frequency allocations, and spiral ganglion place pitch in cochlear implants,” Ear Hear. 36(5), e207–e213. 10.1097/AUD.0000000000000163 25860624 PMC4549170

[46] Levitt, H., and Rabiner, L. R. (1967). “ Use of a sequential strategy in intelligibility testing,” J. Acoust. Soc. Am. 42(3), 609–612. 10.1121/1.1910630 6073974

[45] Levitt, H. C. C. H. (1971). “ Transformed up‐down methods in psychoacoustics,” J. Acoust. Soc. Am. 49(2B), 467–477. 10.1121/1.1912375 5541744

[48] Long, C. J., Nimmo-Smith, I., Baguley, D. M., O'Driscoll, M., Ramsden, R., Otto, S. R., Axon, P. R., and Carlyon, R. P. (2005). “ Optimizing the clinical fit of auditory brain stem implants,” Ear Hear. 26(3), 251–262. 10.1097/00003446-200506000-00002 15937407

[49] Luo, X., Wu, C. C., and Pulling, K. (2021). “ Combining current focusing and steering in a cochlear implant processing strategy,” Int. J. Audiol. 60(3), 232–237. 10.1080/14992027.2020.1822551 32967485 PMC7969432

[50] Macherey, O., and Carlyon, R. P. (2014). “ Re-examining the upper limit of temporal pitch,” J. Acoust. Soc. Am. 136(6), 3186–3199. 10.1121/1.4900917 25480066 PMC4340596

[51] Macherey, O., Carlyon, R. P., Chatron, J., and Roman, S. (2017). “ Effect of pulse polarity on thresholds and on non-monotonic loudness growth in cochlear implant users,” J. Assoc. Res. Otolaryngol. 18, 513–527. 10.1007/s10162-016-0614-4 28138791 PMC5418159

[52] Macherey, O., Deeks, J. M., and Carlyon, R. P. (2011). “ Extending the limits of place and temporal pitch perception in cochlear implant users,” J. Assoc. Res. Otolaryngol. 12, 233–251. 10.1007/s10162-010-0248-x 21116672 PMC3046333

[53] Macherey, O., Van Wieringen, A., Carlyon, R. P., Deeks, J. M., and Wouters, J. (2006). “ Asymmetric pulses in cochlear implants: Effects of pulse shape, polarity, and rate,” J. Assoc. Res. Otolaryngol. 7, 253–266. 10.1007/s10162-006-0040-0 16715356 PMC2504608

[54] McKay, C. M., McDermott, H. J., and Carlyon, R. P. (2000). “ Place and temporal cues in pitch perception: Are they truly independent?,” Acoust. Res. Lett. Online 1(1), 25–30. 10.1121/1.1318742

[55] Mesnildrey, Q., Venail, F., Carlyon, R. P., and Macherey, O. (2020). “ Polarity sensitivity as a potential correlate of neural degeneration in cochlear implant users,” J. Assoc. Res. Otolaryngol. 21, 89–104. 10.1007/s10162-020-00742-7 32020417 PMC7062980

[56] Middlebrooks, J. C., and Snyder, R. L. (2010). “ Selective electrical stimulation of the auditory nerve activates a pathway specialized for high temporal acuity,” J. Neurosci. 30(5), 1937–1946. 10.1523/JNEUROSCI.4949-09.2010 20130202 PMC2828779

[57] Nadol, J. B., Jr. (1990). “ Degeneration of cochlear neurons as seen in the spiral ganglion of man,” Hear. Res. 49(1–3), 141–154. 10.1016/0378-5955(90)90101-T 2292494

[58] Penninger, R. T., Kludt, E., Büchner, A., and Nogueira, W. (2015). “ Stimulating on multiple electrodes can improve temporal pitch perception,” Int. J. Audiol. 54(6), 376–383. 10.3109/14992027.2014.997313 25630393

[59] Rattay, F., Lutter, P., and Felix, H. (2001). “ A model of the electrically excited human cochlear neuron: I. Contribution of neural substructures to the generation and propagation of spikes,” Hear. Res. 153(1-2), 43–63. 10.1016/S0378-5955(00)00256-2 11223296

[60] Resnick, J. M., O'Brien, G. E., and Rubinstein, J. T. (2018). “ Simulated auditory nerve axon demyelination alters sensitivity and response timing to extracellular stimulation,” Hear. Res. 361, 121–137. 10.1016/j.heares.2018.01.014 29496363 PMC5846345

[61] Schuknecht, H. F. (1993). *Pathology of the Ear* ( Lea & Febiger, Philadelphia, PA).

[62] Shannon, R. V., Zeng, F. G., Kamath, V., Wygonski, J., and Ekelid, M. (1995). “ Speech recognition with primarily temporal cues,” Science 270(5234), 303–304. 10.1126/science.270.5234.303 7569981

[63] Spoendlin, H., and Schrott, A. (1989). “ Analysis of the human auditory nerve,” Hear. Res. 43(1), 25–38. 10.1016/0378-5955(89)90056-7 2613564

[64] Stahl, P., Macherey, O., Meunier, S., and Roman, S. (2016). “ Rate discrimination at low pulse rates in normal-hearing and cochlear implant listeners: Influence of intracochlear stimulation site,” J. Acoust. Soc. Am. 139(4), 1578–1591. 10.1121/1.4944564 27106306

[65] Stakhovskaya, O., Sridhar, D., Bonham, B. H., and Leake, P. A. (2007). “ Frequency map for the human cochlear spiral ganglion: Implications for cochlear implants,” J. Assoc. Res. Otolaryngol. 8, 220–233. 10.1007/s10162-007-0076-9 17318276 PMC2394499

[66] Thakkar, T., Kan, A., and Litovsky, R. Y. (2023). “ Lateralization of interaural time differences with mixed rates of stimulation in bilateral cochlear implant listeners,” J. Acoust. Soc. Am. 153(3), 1912–1923. 10.1121/10.0017603 37002065 PMC10036141

[67] Tong, Y. C., Blamey, P. J., Dowell, R. C., and Clark, G. M. (1983). “ Psychophysical studies evaluating the feasibility of a speech processing strategy for a multiple‐channel cochlear implant,” J. Acoust. Soc. Am. 74(1), 73–80. 10.1121/1.389620 6688434

[68] Townshend, B., Cotter, N., Van Compernolle, D., and White, R. L. (1987). “ Pitch perception by cochlear implant subjects,” J. Acoust. Soc. Am. 82(1), 106–115. 10.1121/1.395554 3624633

[69] Trieger, A., Schulze, A., Schneider, M., Zahnert, T., and Mürbe, D. (2011). “ In vivo measurements of the insertion depth of cochlear implant arrays using flat-panel volume computed tomography,” Otol. Neurotol. 32(1), 152–157. 10.1097/MAO.0b013e3181fcf04d 20962701

[70] Venter, P. J., and Hanekom, J. J. (2014). “ Is there a fundamental 300 Hz limit to pulse rate discrimination in cochlear implants?,” J. Assoc. Res. Otolaryngol. 15, 849–866. 10.1007/s10162-014-0468-6 24942704 PMC4164693

[71] Zeng, F. G. (2002). “ Temporal pitch in electric hearing,” Hear. Res. 174(1–2), 101–106. 10.1016/S0378-5955(02)00644-5 12433401

